# Seroprevalence of diphtheria and pertussis immunoglobulin G among children with pneumonia in Ji’nan, China

**DOI:** 10.1186/s12887-018-1337-y

**Published:** 2018-12-05

**Authors:** Qinghong Meng, Lijun Li, Wei Shi, Qing Wang, Mingjie Ding, Yanqin Liu, Xiang Ma, Kaihu Yao

**Affiliations:** 1 Beijing Key Laboratory of Pediatric Respiratory Infection Diseases, Key Laboratory of Major Diseases in Children, Ministry of Education, National Clinical Research Center for Respiratory Diseases, National Key Discipline of Pediatrics (Capital Medical University), Beijing Pediatric Research Institute, Beijing Children’s Hospital, Capital Medical University, National Center for Children’s Health, Beijing, 100045 China; 2 Respiratory department, Qilu Children’s Hospital, Shandong University, Ji’nan, 250022 China

**Keywords:** Pertussis, Pertussis toxin, IgG, Children

## Abstract

**Background:**

Vaccination is still one of the most important methods to control and prevent childhood infections including diphtheria and pertussis. This study evaluated the level of diphtheria (DT) and pertussis (PT)-related antibodies among children with pneumonia in Ji’nan, China.

**Methods:**

A total of 484 sera of children from 1 day to 13 years of age were collected from 2014 to 2015 in Ji’nan. Children with recent history of pertussis were excluded from this study. Anti-DT and PT IgG concentrations were measured by ELISA (Euroimmun, Lübeck, Germany).

**Results:**

Of the 484 subjects tested, the overall positivity rate of anti-DT IgG (≥0.1 IU/ml) was 48.97%, and the highest positivity rate of anti-DT IgG (68.55%) and proportion with long term protection (23.27%) were observed in children aged 6 m- < 3 y. For anti-PT IgG, 334 subjects (69.01%) had anti-PT IgG levels below the lower limit of detection (5 IU/ml). Even with detectable anti-PT antibodies, the majority (115/150, 76.67%) of them had antibody levels of 5- < 40 IU/ml. The highest proportion of subjects with detectable anti-PT IgG (≥5 IU/ml) was observed in children aged < 6 m (44.36%), then the proportion continually decreased to 15.0% at 3 y- < 6 y (*χ*^*2*^ = 24.05, *p* < 0.0001). The highest positivity rate (≥40 IU/ml) was only 8.27% in children aged < 6 m. Subjects with an anti-PT IgG ≥100 IU/ml were observed in all the groups and there were no significant differences in the proportions of subjects with a level ≥ 100 IU/ml among these age groups (*χ*^*2*^ = 2.572, *p* = 0.4624). A total of 5 subjects had anti-PT IgG ≥100 IU/ml (≥1 years post pertussis vaccination) which was considered to be indicative of a recent pertussis infection.

**Conclusions:**

We demonstrated low antibody levels and protection against pertussis in our study population. The anti-PT IgG maintained a low level throughout all age groups, and even no immune responses were observed after the basic immunization and booster. Our study supported the need to reevaluate the immune response of DTP vaccine which was used in Shandong province after 2010.

## Background

Diphtheria and pertussis, caused by *Corynebacterium diphtheria* and *Bordetella pertussis*, are highly infectious and vaccine-preventable respiratory diseases [1, 2]. Diphtheria and pertussis vaccination significantly reduced the incidence of these diseases. However, pertussis has re-emerged in some developed countries that maintain high vaccine coverage [3]. For an example, there was a 50-y high of 48,277 cases of pertussis in the United States in 2012 [4]. In 2012, according to the Pan American Health Organization/World Health Organization (PAHO/WHO) estimation, there were 50 million cases of pertussis which led to 300,000 deaths worldwide [5]. Therefore, pertussis remains one of the leading causes of vaccine-preventable deaths in the world today.

In China, a combined diphtheria-tetanus-whole cell pertussis (DTwP) vaccine was introduced in 1978. From 2007, a combined diphtheria-tetanus-acellular pertussis (DTaP) vaccine was introduced in the Expanded Program on Immunization (EPI) of China, and both DTwP and DTaP were used since then. Only DTaP was administered after 2010 [6, 7]. The first immunization of DTP is administrated in the third month after birth, which is followed by another two dosages in the fourth and fifth month for the basic immunization. The children will be inoculated again for the booster immunization at 18–24 months. After that, the individuals will not get inoculation against pertussis any more in China. DTP vaccination is mandatory, and it is freely available by the public health system. According to official Chinese estimates, the immunization coverage has been over 99% since 2011. No case of diphtheria has been reported in China since 2007 [8]. However, the number of pertussis cases has increased in recent years according to reports from the Center for Disease Control and Prevention (CDC) in China, from 6658 cases in 2015 and 5584 in 2016 to 10,390 in 2017 [9]. In fact, pertussis was most likely underestimated [10], due to the lack of diagnostic testing in most community hospitals in China. In a recent prospective study conducted from 2012 through 2013 in Xi’an, China, more than half of the 313 children who had been coughing for more than 2 weeks were proven to be positive for *B. pertussis* which was determined by nasopharyngeal swab culture and PCR [11]. Therefore, pertussis is still a notifiable disease in children in China.

Vaccination is still one of the most important methods to control and prevent pertussis. Pertussis toxin (PT) is the most specific and protective antigen for pertussis. Both DTwP and DTaP vaccines contain PT. Therefore, anti-PT IgG is a good indicator for surveillance of the effectiveness of DTP vaccines in vaccinated populations. In this present study we evaluated the immune status against pertussis in different age groups of children. The present study included the data of diphtheria and pertussis in children. By comparing the 2 components in the same vaccine - diphtheria and pertussis, it will help us better understand the change of immunity level against them in children.

## Methods

### Serum sample

The sample size of this study was evaluated based on a recent report. A recent seroepidemiological study conducted in Beijing reported that about 15–70% of subjects between the ages of 1–14 years had detectable anti-PT IgG levels [12]. Assuming conservatively a detectable rate of 20% and a significance level of 5%, a minimum number of 94 samples are needed to reach a statistical power of 80%. According to the feasibility and situation of clinical practice, the sample size was expanded to > 400.

From August 2014 to March 2015, 484 children with pneumonia hospitalized in the respiratory department of Qilu Children’s Hospital, Shandong University were included in this study. Those cases diagnosed with pertussis, suspected pertussis and immune system related diseases were not enrolled. In addition, those who had history of coughing ≥2 weeks during the previous year were also excluded in this study. For all children, basic data about age, gender, date of sampling, medical histories and DTP vaccination history were collected from their parents. All serum samples were frozen at − 20 °C until analysis.

### Serological testing

Anti-DT IgG was detected using the commercially available ELISA kits (Euroimmun, Lübeck, Germany). The antibody results were expressed in international units per milli-liter (IU/ml) and referred to the “International Standard for Diphtheria Antiserum”, NIBSC code: 00/496. According to the manufacturer’s instructions, the results were divided to six groups: < 0.01 IU/ml (undetectable/no protection), 0.01- < 0.1 IU/ml (no protection), 0.1–1.0 IU/ml (short term protection/basic protection), > 1.0–1.5 IU/ml (long term protection/booster after 5 years), > 1.5–2.0 IU/ml (long term protection/booster after 7 years) and > 2.0 IU/ml (long term protection/booster after 10 years).

Anti-PT IgG was also detected using the commercially available ELISA kits (Euroimmun, Lübeck, Germany). The antibody results were expressed in international units per milli-liter (IU/ml) and referred to the “First International Standard for Pertussis Antiserum”, NIBSC code: 06/140, World Health Organization (WHO). The lower limit of detection was 5 IU/ml. The anti-PT IgG levels ≥40 IU/ml was categorized as positive, according to the manufacturer’s protocol. Anti-PT IgG ≥100 IU/ml (≥1 years post pertussis vaccination) was considered to be indicative of a recent pertussis infection.

### Quality control

After the samples were tested, we randomly chose 5% of the samples for re-testing. Then, we calculated the coefficient of variation (CV) of the results for these re-tested samples. The results were only considered valid if the CV value of each sample was < 15%, otherwise the test was repeated. Each serological test run was carried out with positives and negative serological controls.

### Data analysis

The study population was divided into four age groups: < 6 m, 6 m- < 3 y, 3 y- < 6 y and ≥ 6 y. For statistical analysis, antibody concentrations below the lower limit for quantitation were assigned as half the lower limit of quantitation (0.005 IU/ml for DT, and 2.5 IU/ml for PT). The value of anti-DT level ≥ 2.0 IU/ml and anti-PT level ≥ 200 IU/ml was counted as 2.0 IU/ml and 200 IU/ml, respectively. Data were analyzed using the GraphPad Prism software (version 5; GraphPad Software, La Jolla, CA, USA) and SPSS (version 19.0). *χ*^*2*^ test was used to compare positivity rate, rate of subjects with long term protection and proportions of subjects with undetectable anti-PT IgG among different age groups. The difference in antibody concentration among different age groups was tested by the Wilcoxon/Krusakal Wallis test. *p* values ≤0.05 were considered statistically significant.

## Results

### Characteristics of study population

A total of 484 subjects were enrolled in the study with the age range of 1 day to 13 y (median: 1.8 y). The ratio of male to female was 2.13:1 (324:160). Among subjects > 3 m, the rate of at least one dose of DTP vaccination was 74.4% (360/406). Among the subjects > 6 m, more than 84.3% (296/351) had a confirmed history of DTP vaccination with at least 3 doses. There were 20, 8 and 1 unvaccinated subjects identified in age groups of < 6 m, 6 m- < 3 y and 3 y- < 6 y, respectively. A total of 17 subjects were unknown for the history of vaccination against diphtheria or pertussis.

### Seroprevalence of anti-DT IgG

**Fig. 1:**
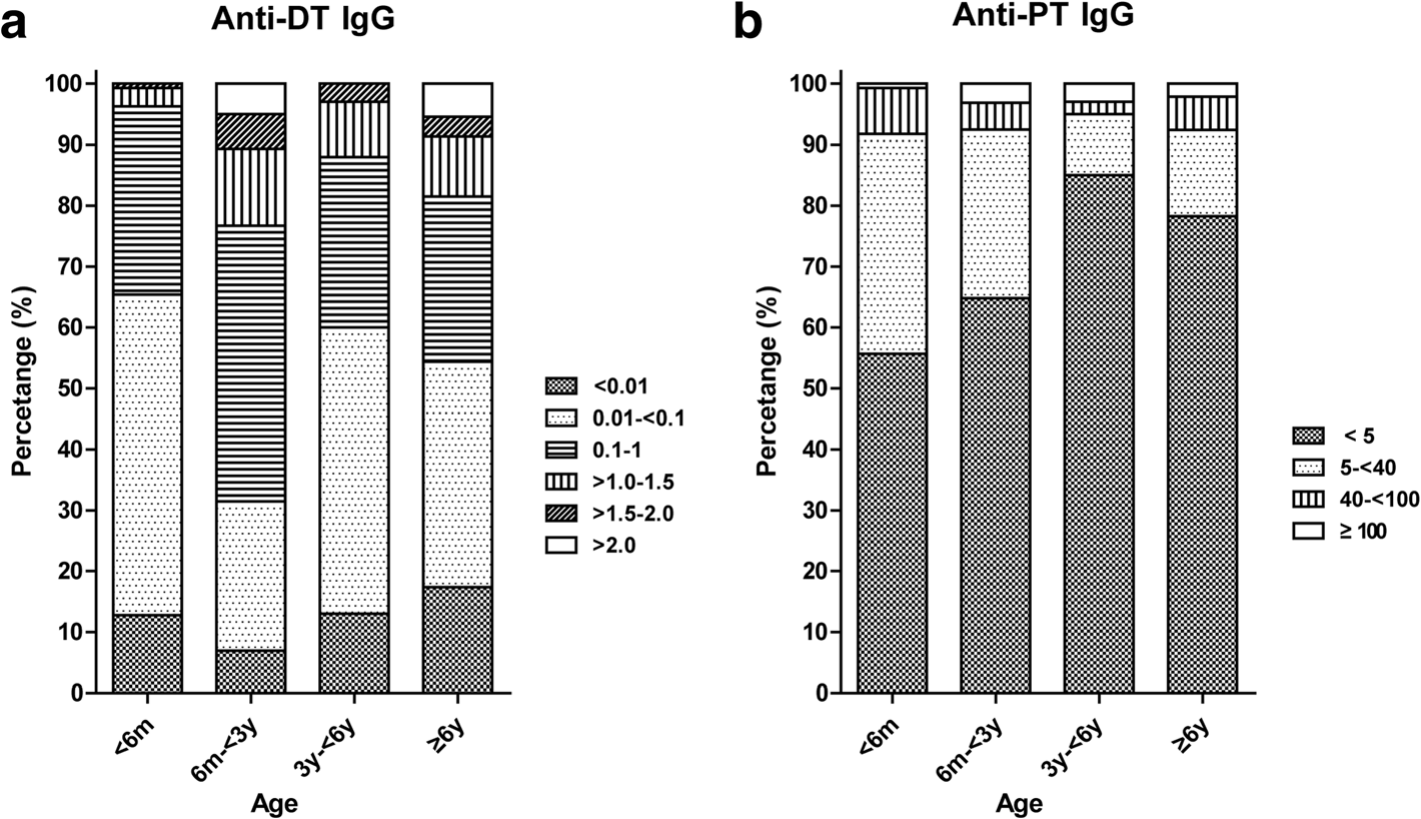
The distribution of anti-DT (**a**) and anti-PT (**b**) IgG level in the sera of subjects among different age groups

The overall positivity rate of anti-DT IgG (≥0.1 IU/ml) was 48.97% with a mean concentration of 0.38 ± 0.53 IU/ml (median: 0.10 IU/ml). There was no significant difference in positivity rates and IgG level between males and females (Table 1). Of the 484 subjects tested, a total of 247 (51.03%) subjects lacked protection, 166 (34.30%) subjects had basic protection, and 71 (14.70%) of them showed long term protection (> 1.0 IU/ml). Fig. 1a shows more details about the distribution of anti-DT IgG in subjects from different age groups. Compared with children without full vaccinations (< 6 m), the positivity rates of anti-DT IgG in the 6 m- < 3 y group (68.55% vs 34.59%, *χ*^*2*^ = 34.162, *p* < 0.0001) increased significantly, while the rate of subjects with long-term protection in the 6 m- < 3 y group also increased (23.27% vs 3.76%, *χ*^*2*^ = 22.639, *p* < 0.0001). Then, the positivity rate and proportion with long term protection at 3 y- < 6 y decreased to 40.0% (*χ*^*2*^ = 20.558, *p* < 0.0001) and 12.0% (*χ*^*2*^ = 5.346, *P* = 0.0208), respectively. After tetanus and reduced diphtheria combined vaccine (Td) booster at 6 y, the proportion of high- level anti-DT IgG (> 2.0 IU/ml) increased significantly (5.43% vs 0%, *χ*^*2*^ = 7.503, *p* = 0.0062). Similar to the patterns of change in the positivity rate of anti-DT IgG, the concentration of anti-DT IgG observed in the 6 m- < 3 y (*p* < 0.0001) group increased significantly (Table 1).

**Table 1:** Demographic and status of anti-DT and anti-PT IgG in the sera of subjects among different age groups

		Anti-DT		Anti-PT	
	N	Positive rate (≥0.1 IU/ml) (95% CI, %)	Concentration and 95% CI (IU/ml)	Positive rate (≥40 IU/ml) (95% CI, %)	Concentration and 95% CI (IU/ml)
Gender					
Male	324	48.15 (42.76–53.58)	0.37 (0.32–0.43)	8.03 (5.53–11.50)	13.44 (9.20–17.67)
Female	160	50.00 (42.34–57.66)	0.40 (0.31–0.49)	5.63 (2.99–10.34)	11.43 (6.32–16.54)
*p*		0.7014	0.4594	0.3376	0.7663
Age					
< 6 m	133	34.59 (27.04–43.0)	0.19 (0.14–0.24)	8.27 (4.68–14.20)	11.76 (7.88–15.65)
6 m- < 3y	159	68.55 (60.97–75.26)	0.56 (0.47–0.66)	7.55 (4.37–12.73)	12.59 (7.72–17.46)
3y- < 6y	100	40.00 (30.94–49.80)	0.62 (0.45–0.79)	5.00 (2.15–11.18)	10.34 (4.02–16.66)
≥6y	92	45.65 (35.88–55.80)	0.44 (0.31–0.57)	7.61 (3.73–14.88)	12.32 (5.47–19.17)
*p*		< 0.0001	< 0.0001	0.7776	< 0.0001
Total	484	48.97 (44.54–53.41)	0.38 (0.33–0.43)	7.23 (5.25–9.89)	11.85 (9.21–14.48)

### Seroprevalence of anti-PT IgG

The anti-PT IgG level in 334 subjects (69.01%) was below the lower limit of detection (5 IU/ml). Even with detectable anti-PT IgG, the majority (115/150, 76.67%) of them had antibody levels of 5- < 40 IU/ml. The mean concentration of anti-PT IgG was 11.85 ± 29.51 IU/ml (median: 2.5 IU/ml) (Table 1). The highest proportion of subjects with anti-PT IgG > 5 IU/ml (44.36%) was observed in children aged < 6 m. Although there was DTP vaccination in the 3rd, 4th and 5th months and booster immunization at 18–24 months, there were no significant differences in the positivity rate among these age groups (*χ*^*2*^ = 1.074, *p* = 0.7834). The highest positivity rate was only 8.27%, observed in children in the < 6 m group. Meanwhile, the proportion of subjects with anti-PT IgG > 5 IU/ml decreased to 15.0% at < 6 y (*χ*^*2*^ = 24.047, *p* < 0.0001). Although there was no DTP booster at 6 y, the proportion of subjects with anti-PT IgG > 5 IU/ml increased (21.74% vs 15.0%, *χ*^*2*^ = 1.461, *p* = 0.2268). There was significant difference (*p* < 0.0001) in concentration of anti-PT IgG among different age groups (Table 1). Compared with children in 6 m- < 3 y group, the concentration of anti-PT IgG observed in the 3 y- < 6 y (*p* < 0.0001) group decreased significantly (*p* = 0.0015). Subjects with an anti-PT IgG ≥100 IU/ml were observed in all the groups, and there were no significant differences in the proportions of subjects with a level ≥ 100 IU/ml among these age groups (*χ*^*2*^ = 2.572, *p* = 0.4624). A total of 5 subjects (3 in the 3 y- < 6 y group and 2 in the ≥6 y group) had an anti-PT IgG ≥100 IU/ml (≥1 years post pertussis vaccination), which was considered to be indicative of a recent pertussis infection (Fig. 1b).

## Discussion

As shown in this study, the increase of anti-DT IgG was consistent with the current immunization schedule in China. However, this population was generally lacking protective immunity to pertussis. The low immunity to *B. pertussis* was associated with an increased risk of pertussis outbreak in Ji’nan. The different patterns of antibody level observed for DT and PT in one combined vaccine indicate the less-than-ideal immune response of anti-pertussis induced by the current DTP vaccine.

In China, the last diphtheria case was recorded in 2006 [8], which means natural boosting by circulating *Corynebacterium diphtheria* occurred less often [13]. Hence, anti-DT IgG levels are almost completely acquired from DTP vaccination. For safety reasons, a tenfold higher value (0.1 IU/ml) than the internationally specified minimum title for prophylaxis was recommended [13–15]. According to the standard, the overall positivity rate of anti-DT IgG in our study was 48.97%. If the lower cut off value (0.01 IU/ml) was adopted [16], the positivity rate of DT-IgG increased to 88.82%. After the implementation of basic immunization at the 3rd, 4th and 5th months of age, there was an increase of the positivity rate and proportions of long term protection observed in the 6 m- < 3 y group. No significant increase in the positivity rate was observed after the 2nd booster immunization at 6 y, however, the proportion with high concentrations (> 2 IU/ml) showed an increase in this age group. Therefore, the results of anti-DT IgG in the study showed that a certain amount of antibody response was generated after Td vaccination.

Much to our surprise, the pattern of anti-pertussis immunity was quite different from diphtheria. The anti-PT IgG maintained a low-level throughout all age groups, and even no immune responses were observed after the basic immunization and booster. According to the current Immunization Program Schedule in China, children had received four doses of DTP vaccines at 2 years of age [17]. The coverage rate of three primary doses of DTwP was more than 90% since 1990, thus, theoretically, subjects 6 m- < 3 y of age should have higher anti-PT IgG than any other age groups. However, about 65.0% of children in this age group showed anti-PT IgG beyond the lower limit of detection (5 IU/ml), and only 3.14% of them had anti-PT IgG levels ≥100 IU/ml after DTP vaccination in 1 year. This pattern of anti-pertussis immunity was similar with that in another previous study carried out in Beijing [12]. As shown in that study in Beijing, even after four doses of DTP vaccination at 2 y, the proportion of subjects with undetectable anti-PT IgG increased gradually in the 3y, 4y, 5y and 6y groups. The highest proportion of subjects with undetectable anti-PT IgG (84.62%) was observed in children aged 7 y. The results of the study indicated that vaccination has limited contribution to the immunity level of pertussis in the population. Another interpretation for no immune responses after the basic immunization and the booster was that the duration of anti-PT IgG after vaccination was very limited [18, 19]. However, more than half of vaccinated children (53.1%) between 6 m–8 m of age had anti-PT IgG levels below the lower limit of detection (5 IU/ml) in our study.

During the preparation of this manuscript, the China Food and Drug Administration reported that the potency indexes of two batches (201605014–01 and 201607050-2) of DTP vaccines produced by two companies did not reach the requirements of antibody titer, on November 3, 2017. The serum samples in our study were collected in 2014–2015, therefore, these two batches had not been used for the children in our study. The highest rate of subjects with undetectable anti-PT IgG indicated less-than-ideal immune response of DTP vaccination in this study. Therefore, the phenomenon about insufficient potency of DTP vaccines may be common. The children < 4 y in this study were born after 2010, and they got DTaP vaccination. The DTwP or DTaP were used in children > 4 y in this study. The low detectable rate of anti-PT IgG among different age groups also indicated insufficient potency of both DTwP and DTaP vaccinations. As the only organization which approved vaccine products used in China, the China Food and Drug Administration should strengthen the supervision and testing of vaccines.

An Estonia study demonstrated that nearly half of subjects < 18 y had undetectable anti-PT IgG levels [20], which were also evaluated with Euroimmun ELISA kits. The author stated that the relatively high proportion of subjects with undetectable anti-PT IgG levels and the relatively low rate of officially reported pertussis cases suggested that low antibody levels did not necessarily indicate an absence of protection. There was also growing evidence that CD4^+^ T cell-mediated cellular immunity was necessary for clearance of *B. pertussis* infection [21]. However, different from the Estonia study, no humoral immune response was observed after the basic immunization and booster. Although anti-PT IgG was not a complete indicator for immunity, the high proportion of children with undetectable anti-PT IgG indicated the less-than-ideal immunogenicity of DTP vaccine.

In our study, 5 subjects (> 3 y) had anti-PT IgG ≥100 IU/ml (≥1 years post pertussis vaccination), indicative of a recent pertussis course. Another 7 subjects had anti-PT IgG 40- < 100 IU/ml (≥1 years post pertussis vaccination), indicative of a possible pertussis course. These cases suggest that this population may be vulnerable. The cases that had diagnosed pertussis and suspected pertussis had been excluded in this study. However, there were cases of recent pertussis infection. Several seroepidemiological studies conducted in different provinces of China also indicated that the incidence of pertussis was most likely underestimated [22, 23]. There are several possible reasons for the pertussis resurgence [24]: (1) increased recognition and reporting of pertussis; (2) diagnostic techniques (such as culture, PCR assay and serologic tests) are available at lots of hospitals; (3) a decrease in vaccine efficacy; (4) antigenic variation of *B. pertussis*. The increase of pertussis cases could be linked to the adverse events of vaccine with insufficient potency, which could become evidence to challenge or deny the effectiveness of vaccination, and bring a persistent inhibition of the public’s enthusiasm for vaccination. Despite the high vulnerability to pertussis, there was no large outbreak of pertussis in China in recent years. To a certain degree, the control strategy against pertussis is still effective at present. Therefore researchers and clinical workers should rationally assess the impact of vaccine titer deficiency on pertussis epidemiology, and improve the public confidence in vaccination. However, there is another possibility. It is usual for people who feel ill to get antibiotics (such as a macrolide) without a prescription in China, which may have efficacy against various bacteria, including *B. pertussis*. This may also explain the serendipitous observation of relatively few pertussis cases among patients who are not well protected by the vaccine.

There were certain limitations in this study. Firstly, one limitation is the restriction of the selected population, since all subjects were pneumonia patients and collected in only one hospital. Multi-center studies in community populations should be performed in other provinces, especially as two batches (201605014–01 and 201607050-2) of DTP vaccines were used. Secondly, there was no definitive cut-off value for single-serum serology in China. Various cut-offs values were used in different studies and countries, such as 30 IU/ml [22], 50 IU/ml [25], 62.5 IU/ml [26], 100 IU/ml [14, 27] and 125 IU/ml [28–30]. According to the manufacturer’s instructions, anti-PT IgG ≥100 IU/ml (≥1 years post pertussis vaccination) was considered to be indicative of a recent pertussis infection. Levels of anti-PT IgG in children were very low in our study. The cut-off value of ≥100 IU/ml might be thus relatively harsh. Thirdly, the history of vaccination was recalled by the parents, which probably resulted in recall bias. The distribution of diphtheria antibody level by age groups also indicated the real proportion of subjects with vaccination could be probably much lower than the estimate of this study.

## Conclusion

We demonstrated low antibody levels and protection against pertussis in our study population. From the epidemiological point of view, this means that these children may be susceptible to pertussis. Our study supported the need to reevaluate the immune response of DTP vaccine which was used in Ji’nan since 2010.

## Abbreviations

CV: Coefficient of variation
DT: Diphtheria
DTaP: Diphtheria-tetanus-acellular pertussis
DTwP: Diphtheria-tetanus-whole cell pertussis
EPI: Expanded program on immunization
IU/ml: International units per milli-liter
PAHO/WHO: Pan American health organization/world health organization
PT: Pertussis
